# Impeding the NHEJ Pathway for Overcoming Radioresistance in the Context of Precision Radiotherapy of Cancer

**DOI:** 10.3390/pharmaceutics18010131

**Published:** 2026-01-20

**Authors:** Dragoș Andrei Niculae, Radu Marian Șerban, Dana Niculae, Doina Drăgănescu

**Affiliations:** 1 Faculty of Pharmacy, University of Medicine and Pharmacy “Carol Davila”, 37 Dionisie Lupu Street, 020021 Bucharest, Romania; 2 Horia Hulubei National Institute for Physics and Nuclear Engineering, 30 Reactorului Street, 077125 Magurele, Romania

**Keywords:** double-strand breaks, DNA repair mechanisms, DNA-PK inhibitors

## Abstract

Non-homologous end joining (NHEJ) is a critical DNA double-strand break (DSB) repair pathway that operates throughout the cell cycle to maintain the genomic stability of the cell. Unlike homologous recombination (HR), NHEJ is capable of repairing DSBs without the need for a homologous template, making it a rapid response mechanism, but potentially prone to errors. Central to NHEJ function and essential for the ligation through the recruitment and activation of additional repair factors, such as Artemis, XRCC4, and DNA ligase IV, is the DNA-dependent protein kinase (DNA-PK) complex. Dysregulation in the NHEJ pathway contributes to genomic instability, oncogenesis, and resistance to genotoxic therapies. Consequently, inhibitors of DNA-PK have emerged as promising therapeutic agents to sensitize tumor cells to radiation and DNA-damaging chemotherapeutics. Inhibiting the DNA-PK ability to recruit the protein complex needed for successful DSB repair promotes cell death through apoptosis or mitotic catastrophe. While inhibitors of DNA-PK can be used to enhance the effects of genotoxic therapies, the field still struggles to address critical problems: how to best exploit the differential DNA repair capacities among tumor subtypes, how to maximize radiosensitization of cancerous cells while sparing normal tissues, and how to translate preclinical studies into clinical benefits. Given that NHEJ constitutes the primary line of defense against radiation-induced damage, rapidly repairing the majority of double-strand breaks throughout the cell cycle, this review concentrates on targeting the DNA-PK complex, as the master regulator of this rapid-response mechanism, highlighting why its inhibition represents a strategic action to overcome intrinsic radioresistance. The implementation of DNA-PK inhibitors into medical practice can enable the stratification of oncologic patients into two categories, based on the tumors’ vulnerability to NHEJ disruptions. Thus, the therapeutic pathways of patients with NHEJ tumors could branch, combining traditional genotoxic therapies (radiation and DNA-damaging chemotherapeutics) with DNA-PK inhibitors to achieve an enhanced effect and improved survival outcomes.

## 1. Introduction

**Figure 1:**
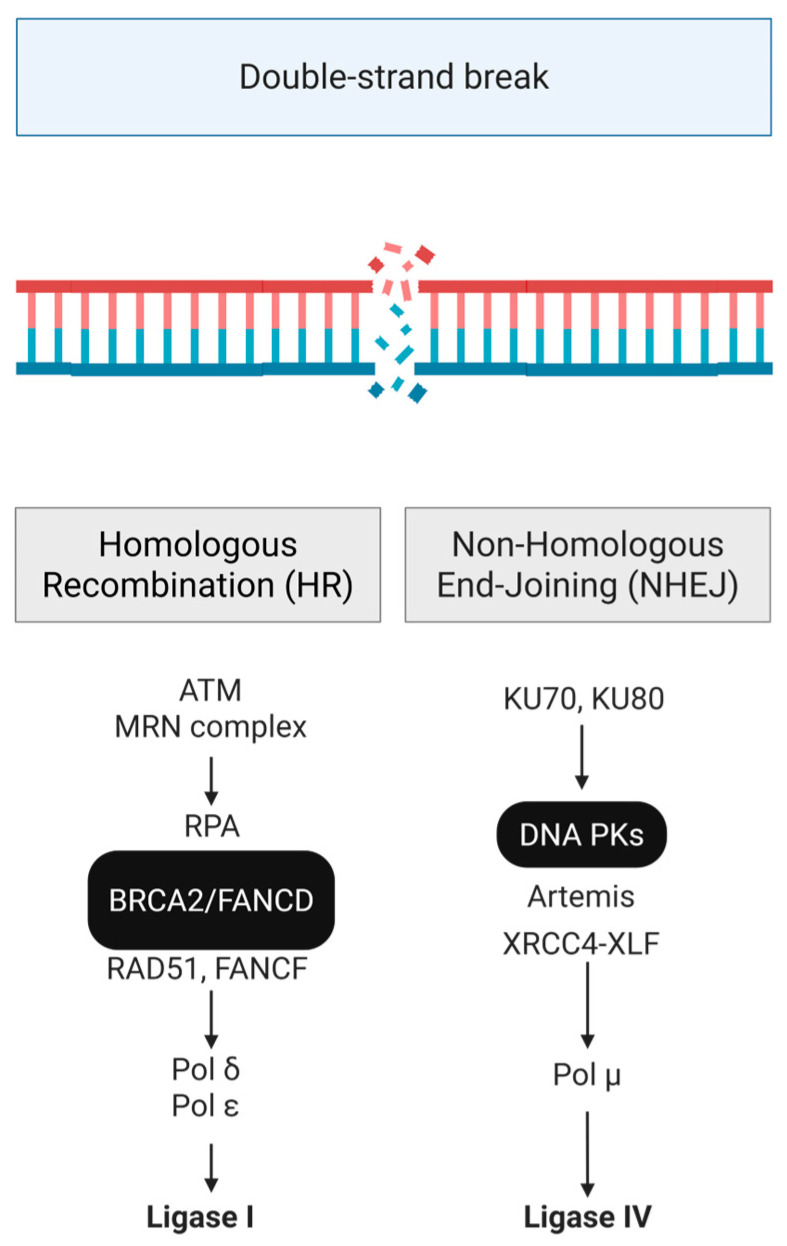
DNA damage response overview: Depending on the type, different mechanisms are employed to recognize and respond to the abnormal structure of genetic material. (Created in BioRender. Niculae, D. (2026). https://BioRender.com/psqzk43).

In the intricate landscape of cellular biology, the reliability of the genome is paramount, safeguarded by a network of DNA repair mechanisms that act as sentinels against genomic instability [1]. This segment will delve into the profound workings of Non-Homologous End Joining (NHEJ), Homologous Recombination (HR), and the DNA-dependent Protein Kinase (DNA-PK) pathway (Figure 1), exploring their roles in maintaining genomic integrity while looking at the interplay of these mechanisms with the mammalian Target of Rapamycin (mTOR) signaling pathway, focusing on their potential regulatory crosstalk.

DNA double-strand breaks (DSBs) are among the most severe forms of DNA damage, involving the simultaneous cleavage of both strands of the DNA helix. These lesions threaten genomic stability and, if not repaired, can cause mutations, chromosomal abnormalities, and cell death—hallmarks of many diseases, including cancer [2].

DSBs arise from both endogenous and exogenous sources. Endogenous factors include reactive oxygen species (ROS) generated during cellular metabolism and the collapse of replication forks. Exogenous sources encompass ionizing radiation (IR), specific chemotherapeutic agents (e.g., topoisomerase inhibitors), and environmental mutagens [3]. Cells have evolved two major repair pathways to manage DSBs: non-homologous end joining and homologous recombination.

NHEJ is the primary repair mechanism in mammalian cells, especially during the G1 phase of the cell cycle when a sister chromatid is unavailable. It involves the direct ligation of DNA ends with minimal or no homology, making it a quick repair method; however, it is potentially prone to errors [4]. Key components of NHEJ include the Ku70/Ku80 heterodimer, DNA-PKcs, XRCC4, and ligase IV.

On the other hand, HR is considered an error-free repair mechanism that uses a homologous DNA sequence, typically the sister chromatid, as a template for repair. It is primarily active during the S and G2 phases. Proteins such as RAD51, BRCA1, BRCA2, and the MRN complex (MRE11-RAD50-NBS1) are crucial for HR, ensuring the high-fidelity restoration of the original DNA sequence [5].

While cancer cells in S and G2 phases have access to the error-free HR pathway, NHEJ remains the primary mechanism for repairing radiation-induced DSBs due to its rapid kinetics. Biological data suggest that the majority of IR-induced breaks are repaired by NHEJ even in late S/G2 phases [6]. Thus, impeding NHEJ effectively sensitizes proliferating tumor cells by disabling the cell’s first line of defence against radiation damage.

The choice between NHEJ and HR is tightly regulated, with proteins like 53BP1 promoting NHEJ and BRCA1 facilitating HR. Dysregulation of DSB repair mechanisms can result in genomic instability. For instance, mutations in BRCA1/2 compromise HR and increase susceptibility to breast and ovarian cancers [7].

Recent advances in genome editing technologies, such as CRISPR-Cas9, exploit the cell’s DSB repair machinery, especially the homology-directed-repair (HDR), an inducible sub-pathway of homologous recombination, to introduce site-specific genetic modifications using an exogenous donor template [8,9]. However, the fidelity of repair remains a concern for clinical applications, emphasizing the need for a deeper understanding of DSB repair dynamics [10].

Thus, DSBs pose a significant challenge to maintaining genome integrity. The complex repair network that has developed underscores the importance of accurate repair for maintaining cellular health and preventing disease. Ongoing research into the molecular details of DSB repair is crucial for advancing cancer therapies [11] and enhancing the accuracy of genome editing technologies [12].

The choice of the DSBs repair pathway depends on the cell cycle phase and the availability of homologous sequences.

In this review, we focus specifically on impeding the NHEJ pathway, with an emphasis on pharmacologic inhibition of DNA-PK, as a strategy to overcome intrinsic and acquired radioresistance in the context of precision radiotherapy (RT) of cancer. We first summarize the major DSB repair pathways and the molecular architecture of NHEJ, including the central role of DNA-PK and its integration with PI3K/AKT/mTOR signalling. We then provide an overview of clinically relevant ATP-competitive DNA-PK inhibitors, highlighting their pharmacologic properties, radiosensitizing activity, and limitations. It is important that the way these agents could be integrated into precision radiotherapy and targeted radionuclide therapy is discussed, with a particular focus on stratifying patients according to NHEJ dependency, normal-tissue tolerance, and mechanisms of resistance.

## 2. DNA Repair Mechanisms

### 2.1. Homologous Recombination

HR is a type of genetic recombination in which nucleotide sequences are exchanged between two similar or identical strands of DNA. This process typically occurs during meiosis in eukaryotic cells, ensuring that chromosomes can pair correctly and exchange genetic material. It is also a key mechanism in prokaryotes for DNA repair and gene integration and is crucial for maintaining genome stability across generations. It is also a contributing factor for genetic diversity during meiosis [13,14,15].

HR is a high-fidelity repair mechanism predominantly active during the S and G2 phases [3]. Initiated by the MRN (Mre11-Rad50-Xrs2 and Sae2 nuclease) complex [16], HR utilizes a homologous DNA template, typically the sister chromatid [17], to mend DSBs with precise fidelity [18]. Following the recognition, exonucleases degrade the 5′ DNA strand, allowing the sister strand to “invade”, processes mediated by the functional BRCA1 and BRCA2 genes [19], and start the strand synthesis by an exact copying process. The recombinase RAD51 plays a pivotal role in facilitating homology search and homologous DNA strand exchange [20], orchestrating the restoration of the damaged DNA sequence. HR is finely regulated to preserve genomic stability and prevent aberrant recombination events [21]. In this review, we refer to HR to indicate the broader double-strand break repair pathway, and HDR when specifically referring to template-directed HR-based repair events as a tool in genome engineering.

External factors such as radiation, certain chemicals, or oxidative stress can cause DNA damage that leads to DSBs [22,23,24]. In response, cells may activate HR pathways to repair these breaks with high fidelity, using a homologous template. In biotechnology and genetic engineering, in order to insert or edit genes with high precision, scientists utilize HDR, an HR-derived process based on precise sequence changes guided by a supplied donor template [25,26].

One of the key advantages of HR is that it enhances genetic diversity during sexual reproduction [15]. Shuffling genetic material between homologous chromosomes helps produce offspring with unique genetic combinations. This diversity is crucial for evolution and adaptation, allowing populations to survive changes in the environment better.

Another significant benefit is its role in accurate DNA repair. HR uses a sister chromatid or a homologous chromosome as a template to repair DSBs precisely. This high-fidelity repair mechanism helps prevent mutations and maintains genome integrity, reducing the risk of genetic diseases or oncogenesis [18]. Furthermore, it was shown that for several tumor phenotypes, such as biliary tract cancers [27], hereditary breast and ovarian cancer [28,29], pancreatic cancer [30], male-breast and prostate cancer [31], the HR pathway is deficient or underexpressed, the loss of which was associated with a higher prevalence of hereditary cancer development.

Despite its benefits, HR can also present drawbacks. One risk is the potential for inappropriate or faulty recombination between non-homologous sequences, which can cause chromosomal rearrangements, deletions, or duplications [32,33]. These errors are linked to genetic disorders and certain cancers, including leukaemia and breast cancer.

In biotechnology, although homologous recombination is a powerful tool for gene editing, it can sometimes be inefficient or cause unintended genetic changes [26]. The process requires precise sequence alignment and can be difficult to control accurately in some organisms. This can create challenges in genetic engineering, especially when working with complex genomes or trying to prevent off-target effects [34].

### 2.2. Non-Homologous End Joining

Conversely, NHEJ, a pivotal mechanism in DSB repair, functions by ligating broken DNA ends with minimal sequence homology [35]. At the core of NHEJ lies a cascade of proteins, including Ku70/Ku80 heterodimers that recognize and bind to DSB ends, and DNA Ligase IV, which finalizes the ligation process [36]. Despite its error-prone nature, NHEJ is swift and operates throughout the cell cycle, providing a rapid response to DNA damage [4].

Recent cryo-electron-microscopy (cryo-EM) reconstructions have resolved the long-range synaptic complex containing Ku70/Ku80, DNA-PKcs, XRCC4–Lig4, XLF, and polymerase λ at 3.6 Å, revealing a ∼47 Å DNA channel and showing that autophosphorylation of DNA-PKcs at the ABCDE and PQR clusters triggers a hinge-like opening that licenses end-processing by Artemis and the Pol X family polymerases [37,38]. Pol λ and Pol μ are now recognized as fidelity modulators: Pol λ promotes accurate fill-in synthesis at compatible ends, whereas Pol μ tolerates mismatches and is recruited when micro-homology is limited, explaining the spectrum of junctional insertions observed in vivo [37]. A second Ku-bound scaffold protein, PAXX, acts redundantly with XLF to stabilize the XRCC4-Lig4 ligation platform; genetic ablation of both PAXX and XLF is synthetically lethal, underscoring their cooperative role in canonical NHEJ (cNHEJ) [39]. Additionally, downstream of 53BP1, the shieldin–CST–Pol α axis counter-balances end resection, channeling breaks away from homologous recombination and toward cNHEJ—a mechanism that becomes critical in BRCA1-deficient tumors and drives PARP-inhibitor resistance [40]. Beyond repair, DNA-PK-dependent chromatin remodeling creates a transient transcription-refractory zone of ~25 kb around each break, mediated by RNAPII eviction and H3K4me3 demethylation, thereby preventing collision between transcription and repair machineries [41]. However, this transcriptional repression is rather selective than absolute. While bulk gene transcription is silenced to avoid conflict, recent studies demonstrate that specific non-coding RNAs, such as damage-induced long non-coding RNAs (dilncRNAs) and DNA damage response RNAs (DDRNAs), are actively generated at DSB sites. These specialized RNA species serve as molecular scaffolds that facilitate the recruitment of key NHEJ factors, including 53BP1 and the Ku70/80 heterodimer, thereby enhancing the repair efficacy within the repressed chromatin environment [42,43].

**Figure 2:**
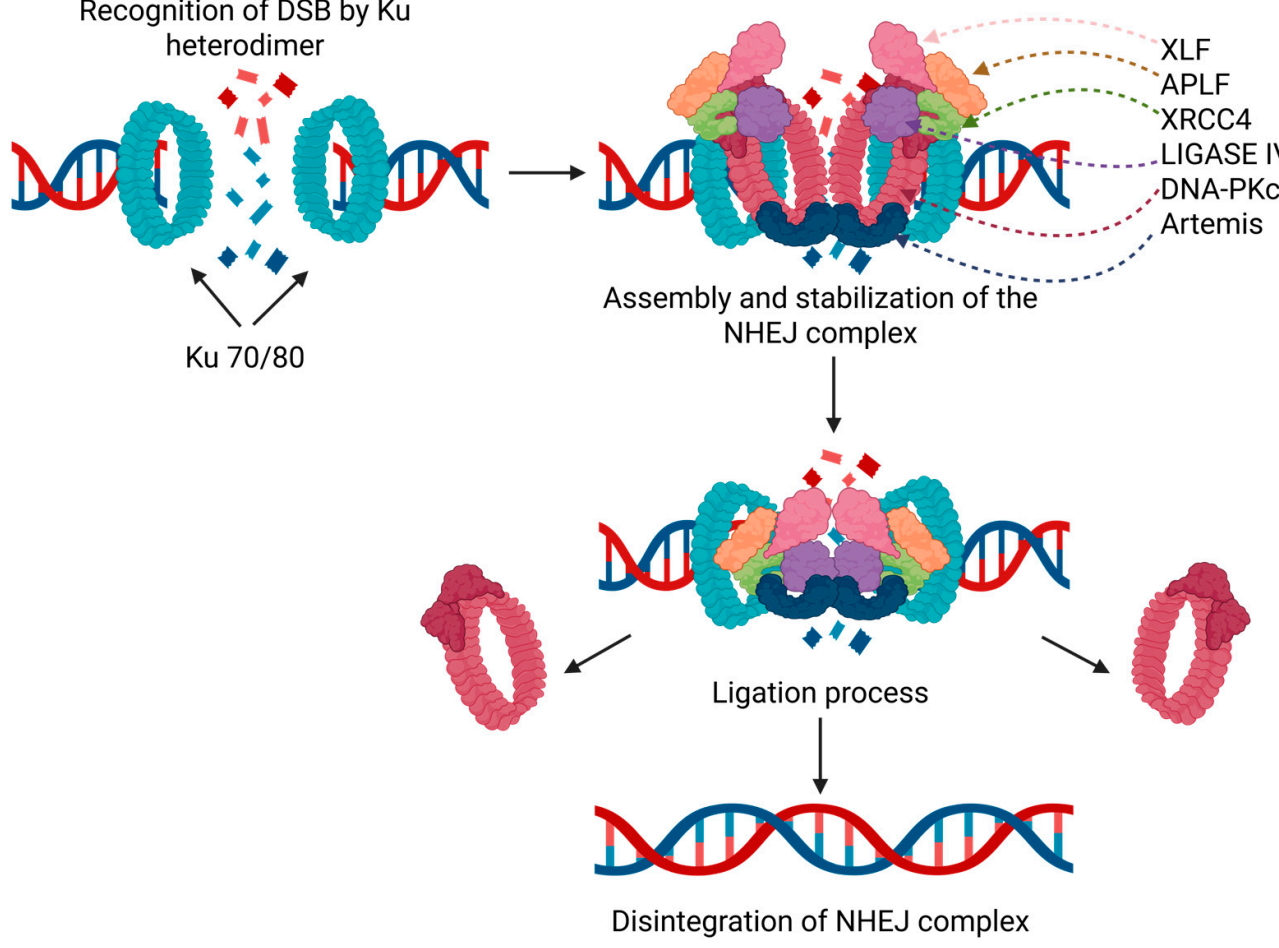
NHEJ repairing-complex mechanism of formation and activity. (Created in BioRender. Niculae, D. (2026). https://BioRender.com/p9ocxx8).

The NHEJ process involves several key steps. Recognition and binding of the Ku70/Ku80 heterodimer to the broken DNA ends, protecting them from degradation and serving as a scaffold for additional proteins; Ku70 is positioned proximal to the DSB point while Ku80 is positioned distal to the DSB [44], forming the initial complex. The recruitment of DNA-PKcs to this complex causes the Ku70/80 to migrate inward [45], thereby creating the DNA-PK holoenzyme. The activation and autophosphorylation of the DNA-PKcs, which enables its kinase function, is essential for downstream processing, including the recruitment and activation of Artemis nuclease, responsible for DNA hairpin opening at coding [46]. It was also shown that DNA-PKcs interaction with Artemis is essential for the repair of many ionizing radiation-induced DSBs [47,48]. The ligation process mediated by the DNA Ligase IV and its cofactor XRCC4, typically in conjunction with XLF, concludes the repair process. Once the repair process is complete, the complex can dissociate (Figure 2). It was shown that higher mRNA expression of ATM and DNA-PKcs was observed in cancer cells as opposed to healthy tissue [49], as well as many cancer types have been associated with higher DNA-PK expression, which, in turn, is correlated with a poorer prognosis [50,51].

It was suggested that NHEJ is a more significant DSB repair mechanism in radioresistant cancer cells than its counterpart, the HR pathway [52], underlining the importance of fully understanding and utilizing this sensitizing potential. Mechanistically, this NHEJ leaning reflects both the kinetic advantage of NHEJ and tumor-specific rewiring of the DSB response. IR-induced breaks are bound by Ku70/Ku80 and DNA-PKcs within minutes, whereas HR requires extensive 5′–3′ end resection and operates on a much slower timescale. Radioresistant tumors frequently show overexpression or hyperactivation of core NHEJ components such as Ku70/Ku80 and DNA-PKcs, as well as end-protection factors downstream of 53BP1 (e.g., shieldin) [53,54], which limit resection and actively channel DSBs into canonical NHEJ rather than HR [55]. Clinically, high Ku70/DNA-PKcs expression correlates with poor local control after radiotherapy, while pharmacological DNA-PK inhibition preferentially radiosensitizes radioresistant tumor models, supporting a causal role for NHEJ proficiency in radioresistance [56].

#### 2.2.1. Integration with mTOR Signaling

The mTOR pathway, a master regulator of cellular homeostasis, has emerged as a pivotal player in the DNA damage response (DDR) [57,58]. mTOR regulates cell growth, proliferation, and survival, and its dysregulation is associated with various pathologies, including cancer. Acting as a sensor of nutrients, growth factors, and energy status, mTOR orchestrates protein synthesis, autophagy, and lipid metabolism. It exists in two distinct complexes: mTOR Complex 1 (mTORC1) and mTOR Complex 2 (mTORC2) [59], each with unique functions and downstream targets [60]. Increasing evidence indicates that mTOR intersects with DNA repair pathways, modulating cellular outcomes after genotoxic stress [57,58,61].

Numerous signaling pathways tightly regulate the activation of mTOR. In response to growth factors, mTORC1 is activated through the Phosphoinositide 3-kinase (PI3K)-Protein kinase B (AKT) pathway and relieved from Tuberous sclerosis 1-2 (TSC1–TSC2) inhibition via AKT-dependent phosphorylation [62]. Amino acids, particularly leucine, further stimulate mTORC1 activation, which then phosphorylates S6 kinase beta (S6K) and Eukaryotic translation initiation factor 4E-binding protein 1 (4E-BP1) to promote mRNA translation and cell-cycle progression [60,63]. Functionally within the DDR, mTORC1/S6K signaling can regulate DNA repair/checkpoint proteins (e.g., S6K-dependent control of Cdk1 and MSH6) [63]. mTORC2, activated by growth factors, phosphorylates AKT at Ser473 and can interface with DNA repair via the AKT–DNA-PK axis [64], prompting cell survival and proliferation [65].

Hyperactive mTOR—especially mTORC1—drives proliferation, angiogenesis, and metabolic reprogramming, and can enhance DNA repair capacity, contributing to tumorigenesis and fostering radio- and chemoresistance [57,61,66]. Overactive mTOR signaling has been associated with numerous cancers—such as breast, prostate, lung, liver, and renal carcinomas—making it an attractive target for anticancer therapies [67,68]. Conversely, reduced mTOR activity disrupts growth and survival programs, effectively mimicking a state of systemic nutrient deprivation. This chronic suppression dampens the anabolic processes required for stem cell differentiation and immune cell clonal expansion, thereby impairing tissue regeneration and immune surveillance [69]. This creates a paradoxical environment where the inhibition intended to arrest tumor growth may weaken the organism’s intrinsic anti-tumor immunity while accelerating aging-associated phenotypes, such as senescence. Therefore, reduced mTOR activity is linked to neurodegeneration [60,70] and metabolic disorders, such as β-cell failure, insulin secretion defects, lipodystrophy, and non-alcoholic fatty liver disease [71,72,73,74], that are ultimately associated with higher cancer incidence and worse prognosis. Insulin resistance and hyperinsulinemia accentuate insulin-like growth factor 1 (IGF-1) and insulin signaling. This activates PI3K–AKT–mTORC1, enhancing anabolism and tumor cell survival [75], while type-2 diabetes is often associated with increased colorectal cancer risk [76]. Moreover, obesity related to type-2 diabetes creates a chronic low-grade inflammatory environment that promotes oncogenesis and progression of the disease [75]. Adipokine dysregulation, usually associated with lower adiponectin and higher leptin, links to higher cancer risk and recurrence in several situations [77,78,79]. It was shown that pre-existing diabetes is linked to poorer survival and suboptimal therapy delivery, while obesity often predicts worse overall survival/disease-free survival [80,81,82].

The inhibition of mTOR has attracted considerable attention as a therapeutic approach, especially in cancer treatment. Rapamycin and its analogs (rapalogs), collectively known as mTOR inhibitors, work by forming a complex with the FK506-binding protein 12 (FKBP12), which binds to the FRB domain to inhibit mTORC1 [83] selectively. Reported outcomes include:

- Antiproliferative effects—mTOR inhibitors impede cell cycle progression and protein synthesis, exerting antiproliferative effects on cancer cells by blocking G1→S translation programs and tumor growth [66,84];
- Induction of autophagy—relieving mTORC1’s brake on autophagy to clear damaged organelles (including mitophagy after genotoxic stress) [70,85,86,87];
- Angiogenesis inhibition—via HIF-1α/VEGF down-modulation and endothelial mTOR blockade, mTOR inhibitors can interfere with angiogenesis, limiting the blood supply to tumors [88,89,90,91,92];
- Immunosuppression—mTOR inhibitors have immunosuppressive properties and are clinically exploited in transplantation to prevent organ rejection [93].

Clinically and preclinically, mTOR inhibition can radiosensitize tumors, reducing DNA repair and altering checkpoint responses; combinations with IR and radioligand therapy are under active study [61,94].

#### 2.2.2. PI3K Pathway

The Phosphoinositide 3-kinase (PI3K) pathway is a crucial cellular signaling pathway that regulates various cellular processes, including cell growth, survival, proliferation, metabolism, and motility [95]. It is activated in response to external signals, such as growth factors, playing a pivotal role in transducing these signals to the intracellular machinery. Dysregulation of this pathway is commonly associated with cancer [96]. The strong connection between the mTOR and PI3K/Akt pathways effectively makes them a single, vital pathway that interacts with key cell regulators such as hypoxia-inducible factors (HIFs), c-Jun N-terminal kinase (JNK), mitogen-activated protein kinase [97], and DNA-PK [95].

External signals, often initiated by growth factors such as IGF or epidermal growth factor (EGF), bind to their respective cell surface receptors (e.g., the insulin receptor, IGF-1R, or epidermal growth factor receptor), thereby initiating the activation step. This binding of the ligand induces the dimerization and activation of the receptor tyrosine kinase (RTK), resulting in the autophosphorylation of tyrosine residues on the receptor [98]. This activated RTK, in turn, recruits and activates PI3K enzymes [99]. Class I PI3Ks phosphorylate the lipid phosphatidylinositol 4,5-bisphosphate (PIP2) at the plasma membrane, converting it to phosphatidylinositol 3,4,5-trisphosphate (PIP3) [100]. PIP3 serves as a second messenger and recruits the serine/threonine kinase Akt [101] (also known as Protein Kinase B, PKB) to the plasma membrane through its pleckstrin homology (PH/PHIP) domain [102]. Akt is then phosphorylated and activated by 3-phosphoinositide-dependent kinase 1 (PDK1) and mammalian target of rapamycin complex 2 (mTORC2). Moving downstream, the activated Akt phosphorylates a multitude of targets, including TSC2 and PRAS40, relieving suppression of mTORC1, BAD and FOXO to enhance cell survival, and AS160/TBC1D4 to increase GLUT4 translocation and glucose uptake. Inactivation of GSK3α/β promotes glycogen synthesis and cell-cycle progression. Collectively, these events couple growth-factor signaling to protein synthesis, metabolism, survival, and proliferation. In the context of genotoxic stress. Akt further supports DNA repair—for example, by promoting non-homologous end-joining via DNA-PKcs—thereby contributing to radio- and chemoresistance when aberrantly activated [103].

#### 2.2.3. DNA-Dependent Protein Kinase (DNA-PK) Pathway

The DNA-PK pathway is an integral component of the NHEJ repair machinery [104], as the DNA-PK holoenzyme, a serine/threonine protein kinase (Ku70/Ku80 heterodimer and the catalytic subunit DNA-PKcs), orchestrates the recognition and initial processing of DNA ends at DSB sites. DNA-PKcs, the largest out of the phosphatidylinositol-3-kinase-related protein kinase family (PIKK) (460 kD), encoded by the PRKDC/XRCC7 gene [105], also phosphorylates key substrates to facilitate subsequent repair steps. This pathway’s complex role extends beyond NHEJ, influencing cellular processes such as transcription, chromatin remodeling, and apoptosis.

The mechanism of recognizing and repairing DSBs is fast and robust. In the first step, the Ku70/Ku80 heterodimer recognizes the broken DNA ends, forming a complex with DNA-PKcs that stabilizes the DNA strands in proximity to one another, making the repair process possible. Secondly, upon binding, DNA-PKcs becomes autophosphorylated, leading to its activation. This autophosphorylation is a crucial step in initiating the NHEJ pathway. Subsequently, DNA-PKcs phosphorylates various substrates involved in end processing and other repair steps, facilitating the recruitment of additional NHEJ factors. Lastly, the final step consists of the ligation of the broken DNA ends by the DNA Ligase IV-XRCC4 complex, ensuring the restoration of genomic integrity.

DNA-PK activation is primarily triggered by the presence of DNA damage, particularly DSBs. The Ku70/Ku80 heterodimer has a high affinity for DNA ends, and its recruitment to the site of damage is an early event in the cellular response to DNA breaks.

Elevated levels of DNA-PK have been associated with increased DNA repair capacity, due to enhanced NHEJ activity [106]. In specific contexts, such as cancer biology, this may contribute to resistance to DNA-damaging therapies. Overactive DNA-PK has been observed in various cancer types, potentially influencing tumor cell survival and resistance to treatment.

**Figure 3:**
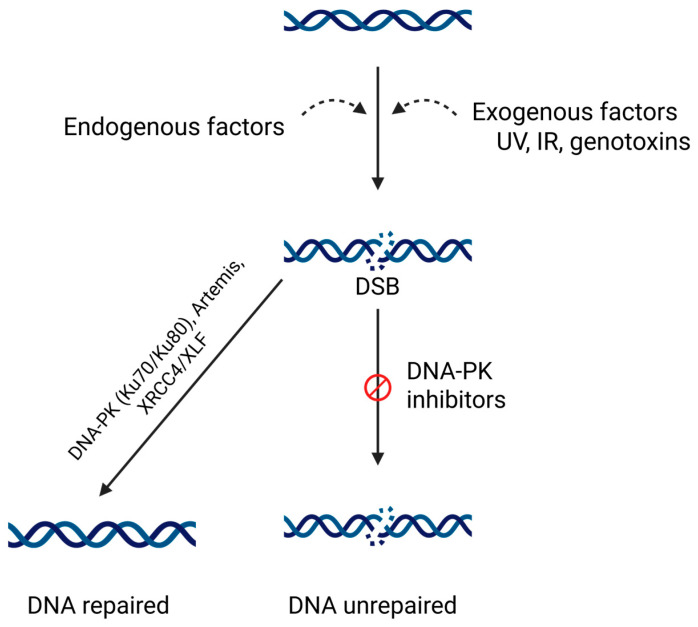
DNA-PK inhibitors preventing damaged DNA repairing, by preventing the K-70/80 recruitment of DNA-PK. (Created in BioRender. Niculae, D. (2026). https://BioRender.com/s03i48c).

Equally, reduced DNA-PK activity can lead to impaired DNA repair, rendering cells more susceptible to genomic instability and mutagenesis. Deficiencies in DNA-PKcs have been linked to severe combined immunodeficiency, a condition characterized by impaired immune function and increased sensitivity to DNA-damaging agents [107]. Inhibition of DNA-PK has gained attention as a potential strategy for cancer treatment, particularly in combination with DNA-damaging agents. Small molecules, such as DNA-PK inhibitors, interfere with the kinase activity of DNA-PKcs, thus inactivating the kinase complex and activity (Figure 3). We will concentrate on these further.

DNA-PK inhibition sensitizes cancer cells to intense DNA-damaging agents like radiation and specific chemotherapeutic agents by impairing the repair of DSBs, enhancing the efficacy of these treatments. DNA-PK inhibitors have also shown promise as antitumor agents by selectively targeting cancer cells with compromised DNA repair mechanisms, while sparing normal cells with intact repair pathways [108,109].

## 3. DNA-PK Inhibitors

Over the years, numerous substances have been investigated for their ability to inhibit DNA-PK activity and prevent the repair of DSBs. Table 1 highlights a selection of relevant DNA-PK inhibitors, their structure, and properties.

### 3.1. NU7441 (KU-57788)

NU7441 is an imidopiperidine derivative that selectively and strongly inhibits DNA-PK by targeting its ATP-binding site, effectively blocking its kinase activity with an IC50 around 14 nM. This effectively impairs the NHEJ DNA repair pathway [110]. It competes with ATP at the catalytic domain of DNA-PKcs, preventing kinase activity [111]. Preclinical studies show NU7441’s potential to sensitize cancer cells to radiation [99,112] and chemotherapy [113,114]. Further research indicates NU7441 induces a sustained G2/M checkpoint, prolongs γH2AX foci, and enhances the effects of photon irradiation in orthotopic hepatocellular and prostate xenografts, achieving dose-enhancement factors of 1.6–1.9 at 10 μM [115]. However, rapid CYP3A4 oxidation and low water solubility limit its in vivo exposure. As a result, current medicinal chemistry efforts use NU7441 as a model for designing second-generation analogues with better pharmacokinetics [115].

### 3.2. M3814—Peposertib

M3814 is a powerful and selective inhibitor of DNA-PK, targeting the ATP-binding pocket of DNA-PKcs. It blocks both the kinase activity and the autophosphorylation necessary for activation. M3814 has progressed to early clinical trials. Phase I studies have evaluated its safety, pharmacokinetics, and initial effectiveness when combined with radiotherapy and other agents that cause DNA damage. In the first human dose-escalation trial (NCT02316197), peposertib achieved substantial target suppression (over 90% reduction in phospho-DNA-PKcs) at 200 mg once daily, with manageable grade 1/2 gastrointestinal side effects; objective responses were mainly seen in Ataxia-Telangiectasia Mutated (ATM)-deficient tumors, indicating a synthetic-lethal relationship [116]. When combined with palliative radiotherapy (30 Gy/10 fractions) in patients with solid head-and-neck tumors, with or without Cisplatin, the combination was tolerable and demonstrated a manageable safety profile with promising efficacy. This efficacy signal, achieved without a significant pharmacokinetic interaction. This lack of interaction is clinically relevant, as it ensures that radiation does not alter the drug’s metabolism or clearance, allowing for predictable dosing without the risk of unexpected systemic toxicity. This, in turn, indicates that the observed benefit is driven by true pharmacodynamic radiosensitization—specifically, the blockade of NHEJ-mediated repair in tumor cells—rather than an artefact of altered drug exposure [117].

### 3.3. CC-115

CC-115 is a dual inhibitor that targets both DNA-PK and mTOR. It blocks the kinase activity of DNA-PK, hindering its role in DNA repair, and also affects the mTOR pathway. CC-115 is currently in clinical trials, mainly for advanced solid tumors and lymphomas. Its dual action makes it a promising candidate for exploring the combined effects of DNA-PK and mTOR inhibition. The phase-I human trial (NCT01353625) in humans with advanced solid and hematological malignancies identified 10 mg BID orally as the recommended dose for phase-II. Dose-limiting toxicities included thrombocytopenia, increased transaminases and hyperglycemia, consistent with mTOR effects. Another recent study shows that for metastatic castration-resistant prostate cancer, when combined with enzalutamide, it reduced prostate-specific antigen (PSA) level [118]. Importantly, CC-115 crosses the blood–brain barrier, with a tumor-to-plasma ratio of about 0.71 in glioblastoma samples. Remarkably, one patient even achieved full regression of endometrial cancer that had been sustained for 4 years [119]. Preclinical models of NSCLC show that CC-115 induces ROS-mediated apoptosis and is more effective than combining single-agent DNA-PK and mTOR inhibitors, suggesting additional redox-related synthetic lethality [120]. These findings indicate pharmacodynamic synergy but so far, early-phase clinical data do not demonstrate that CC-115-based combinations permit substantial dose reductions or clearly fewer adverse events compared with single-pathway inhibition; dosing is still limited by class-typical toxicities of mTOR blockade [119], (119 de mai sus). Recent oncology reviews rank CC-115 among the most advanced DNA-PK agents in clinical development [116,121].

### 3.4. AZD7648

AZD7648 is an orally available, highly selective DNA-PK inhibitor that targets the ATP-binding site of DNA-PKcs. It was discovered through screening and further optimized for selectivity and pharmacokinetic features, reaching an IC_50_ of 0.6 nM against DNA-PK in vitro, with limited off-target effects on other kinases [122,123].

The mechanism of action of AZD7648 involves binding to the ATP-binding site of DNA-PKcs, the catalytic subunit of DNA-PK. This binding prevents the autophosphorylation of DNA-PKcs at Serine 2056, a critical step in NHEJ-mediated DNA repair. Structural studies have shown that AZD7648 fits into a deep hydrophobic pocket within DNA-PKcs, forming stabilizing interactions that enhance its inhibitory potency. By impeding DNA repair, AZD7648 increases the accumulation of DNA damage in cancer cells, leading to enhanced cell death, particularly when used in combination with other therapies [124].

Preclinical studies have demonstrated the efficacy of AZD7648 in combination with various DNA-damaging agents. In vitro, AZD7648 has been shown to potentiate the effects of ionizing radiation and doxorubicin, leading to increased cell death in cancer cell lines. In vivo, combination therapy with AZD7648 and radiation resulted in significant tumor regression in xenograft models, with some models showing up to 84% tumor regression. Additionally, AZD7648 has been found to enhance the efficacy of the PARP inhibitor olaparib, particularly in cells deficient in ATM, a key protein in the DNA damage response [123,125].

The therapeutic potential of AZD7648 goes beyond its cytotoxic effects. When combined with radiation, AZD7648 has been shown to trigger a type I interferon (IFN) response, which can activate the immune system to recognize and attack tumor cells. This immunogenic effect indicates that AZD7648 may not only boost the direct killing ability of DNA-damaging agents but also help develop immunological memory, potentially leading to better long-term tumor control [126].

AZD7648 has entered early-phase clinical trials to evaluate its safety, tolerability, pharmacokinetics, and pharmacodynamics, both as a monotherapy and in combination with other agents. Chemistry optimization yielded a Ki of 0.6 nM, demonstrating greater than 100-fold selectivity over PI3Kα, along with favorable microsomal stability. In xenografts, combining AZD7648 (50 mg/kg once daily) with doxorubicin resulted in sustained regressions and clearance of Circulating Tumor DNA (ctDNA) [122]. The Phase I/IIa study (NCT03907969) reported gastrointestinal dose-limiting toxicities at ≥30 mg when given with pegylated liposomal doxorubicin; nevertheless, pharmacodynamic biopsies confirmed >80% pDNA-PKcs inhibition at plasma trough levels [127].

Currently, AZD7648 is undergoing clinical evaluation in Phase 1 and 2 trials to assess its safety, pharmacokinetics, and efficacy as both monotherapy and in combination with other cancer therapies, such as doxorubicin and olaparib. Given its potent inhibitory activity, selectivity, and promising preclinical results, AZD7648 holds significant promise as a therapeutic agent in the treatment of various cancers. Future studies will be crucial for determining its optimal clinical application and elucidating its role in cancer therapy further [124,128].

### 3.5. VX-984 (M9831)

VX-984, also known as M9831, is a potent and selective DNA-PK inhibitor that hinders the kinase activity of DNA-PKcs, affecting its role in NHEJ. Through its inhibitory action, VX-984 disrupts DSB repair, leading to increased DNA damage and enhanced sensitivity to genotoxic therapies. This mechanism positions VX-984 as a promising candidate for cancer treatment, particularly in tumors with high DNA-PKcs activity [108,129].

The action of VX-984 is characterized by its ability to inhibit DNA-PKcs autophosphorylation, a critical step in the NHEJ repair process. In preclinical studies, VX-984 has been shown to enhance the cytotoxic effects of ionizing radiation and chemotherapy agents by preventing the repair of radiation-induced DSBs. For instance, in glioblastoma cell lines such as U251 and NSC11, VX-984 treatment led to a significant increase in γH2AX foci and a reduction in DSB repair, indicating impaired DNA repair mechanisms [129].

Clinically, VX-984 has demonstrated radiosensitizing effects across various cancer models. In non-small cell lung cancer (NSCLC) xenografts, the combination of VX-984 and IR resulted in durable complete responses, whereas IR alone only delayed tumor growth. VX-984 crosses the blood–brain barrier; in orthotopic glioblastoma models a single 25 mg/kg dose inhibited radiation-induced pDNA-PKcs for >4 h and improved median survival from 29 to 43 days when combined with 6 Gy fractionated radiotherapy [129]. Class-switch recombination and EJ-DR reporter assays confirm that VX-984 suppresses NHEJ without affecting homologous recombination, indicating pathway-selective inhibition that may spare normal tissues with high HR capacity [108]. These findings underscore the potential of VX-984 to augment the therapeutic efficacy of conventional cancer treatments [108].

Currently, VX-984 is undergoing clinical evaluation in Phase 1 and 2 trials, both as a monotherapy and in combination with other therapeutic agents, such as doxorubicin. These studies aim to assess the safety, pharmacokinetics, and efficacy of VX-984 in various types of cancer [124]

### 3.6. PIK-75 HCl

PIK-75 HCl inhibits the catalytic activity of PI3K, specifically the p110α isoform. Despite being developed as a PI3Kα inhibitor, biochemical profiling revealed nanomolar potency against DNA-PK (IC_50_ ≈ 2 nM) and p38γ, designating PIK-75 as a multikinase agent. Formulating PIK-75 into HDL-mimetic nanoparticles overcomes its poor solubility, achieves tumor-targeted delivery via SR-B1, and induces rapid apoptosis in prostate cancer and cutaneous T-cell lymphoma xenografts at 5 mg/kg [130]. By doing so, it interferes with the PI3K/AKT/mTOR signaling pathway, which is frequently dysregulated in various cancers. The dual PI3K/DNA-PK activity may be therapeutically advantageous in MYC-driven tumors, where simultaneous blockade of survival signaling and DSB repair produces synthetic lethality, although clinical translation remains pending. Its ability to interfere with the PI3K pathway makes it a potential candidate for targeted cancer therapy [131,132].

Mechanistically, PIK-75 binds to the ATP-binding site of p110α, inhibiting its kinase activity. This leads to decreased phosphorylation of downstream effectors, such as Akt (Ser473 and Thr308), 4E-BP1, and RPS6, thereby disrupting the PI3K/Akt/mTOR signaling pathways. Inhibition of these pathways impairs cell proliferation and survival, inducing apoptosis in various cancer cell lines [133,134].

In preclinical studies, PIK-75 has demonstrated significant anti-cancer activity. In acute myeloid leukemia (AML) cell lines and primary patient samples, PIK-75 inhibited cell proliferation and induced apoptosis, with IC_50_ values ranging from 62 nM to 173 nM [135]. Significantly, it did not affect normal hematopoietic progenitor cells, indicating a favorable therapeutic index. In glioma models, PIK-75 induced apoptosis and G2/M cell cycle arrest, particularly in PTEN wild-type cells, highlighting its potential in treating glioblastoma [131].

Beyond its anti-cancer properties, PIK-75 exhibits anti-inflammatory effects. It suppresses the production of pro-inflammatory cytokines, such as TNF-α and IL-6, by inhibiting NF-κB activation. In murine models of colitis, PIK-75 administration led to significant suppression of histological abnormalities, suggesting its potential in treating inflammatory diseases [136].

Currently, PIK-75 is being investigated in various preclinical models for its efficacy in combination with other therapies. For instance, in mantle cell lymphoma models, PIK-75 has been shown to overcome venetoclax resistance by inhibiting the PI3K/Akt pathway and downregulating MCL-1 expression. These findings support the continued exploration of PIK-75 as a therapeutic agent in cancer and inflammatory diseases [137].

Although NU7441, peposertib (M3814), CC-115, AZD7648, VX-984 and PIK-75 all bind the ATP-binding domain of DNA-PKcs, they exhibit markedly different biological profiles. This variability reflects several layers of diversity: (i) kinase selectivity and off-target binding, with earlier compounds such as NU7441 and PIK-75 retaining appreciable activity against other PI3K family members or additional kinases, whereas newer agents (M3814, AZD7648, VX-984) were optimized for high DNA-PK selectivity; (ii) pharmacokinetics and pharmacodynamics, including different oral bioavailabilities, microsomal stability, half-life and tissue penetration (e.g., blood–brain barrier penetration by CC-115 and VX-984) [119,129], which determine the depth and duration of target inhibition in tumors; and (iii) tumor-intrinsic biology, as the magnitude of radiosensitization or single-agent activity depends on factors such as ATM or p53 loss, homologous recombination deficiency, mTOR/PI3K pathway hyperactivation, and overall dependence on NHEJ [115,123,125,138]. All in all, these features explain why ATP-competitive DNA-PK inhibitors with similar in vitro potency can produce distinct radiosensitization profiles, toxicity profiles and different degrees of synthetic lethality across distinct tumor profiles.

## 4. Radiosensitization

Radiosensitization encompasses strategies that enhance the cytotoxic effects of ionizing radiation on tumor cells to a greater extent than on surrounding healthy tissue, thereby increasing the tumor-to-normal tissue response and improving local control and the therapeutic index. Because radiotherapy response is shaped by tumor-intrinsic factors (e.g., DNA repair capacity, cell-cycle control) and extrinsic factors (e.g., oxygenation, vasculature, immune contexture), radiosensitizers exploit these vulnerabilities to widen the therapeutic window [139].

Hypoxia is a dominant determinant of radioresistance [140,141]. Mechanistically, the oxygen fixation hypothesis explains that molecular oxygen stabilizes radiation-induced DNA radicals, converting them into non-restorable lesions; in hypoxia, chemical restitution prevails, these radicals are chemically restored by repair mechanisms, thus reducing the biological effectiveness of radiation, and the oxygen-enhancement ratio of ~2.5–3 has been documented [142,143,144]. Clinical meta-analyses show that hypoxia modification improves locoregional control and survival in head-and-neck radiotherapy [145]. Approaches to address microenvironment hypoxia include:

- Hypoxia-activated prodrugs such as evofosfamide (TH-302), which preferentially release cytotoxins under low oxygenation status and have been tested alone and with RT [146,147];
- Physiological modulation through hyperbaric oxygen, carbogen breathing, or nicotinamide to improve reoxygenation [148];
- Vascular normalization approaches, using anti-angiogenic or vasoactive agents to enhance perfusion and reduce hypoxia transiently [149,150,151].

Alternatively, high-atomic-number (high-Z) nanoparticles (e.g., gold, hafnium oxide, platinum) can increase local dose deposition and ROS yield during irradiation by enhancing local photoelectric absorption and secondary electron generation. They can also be functionalized for tumor targeting [152]. This results in augmented ROS production and DNA damage [153]. Functionalization of nanoparticles with targeting ligands or therapeutic payloads can further improve tumor specificity and mitigate systemic toxicity. Preclinical and clinical data include radiosensitization studies with gold nanoparticles on triple-negative breast cancer cell lines [154] and NBTXR3 (hafnium oxide) combined with RT in soft-tissue sarcoma (randomized phase II–III) [155,156,157].

Limitations relevant to translation include low tumor delivery efficiency, variable biodistribution/clearance, and long-term safety questions due to potential accumulation in non-target organs, as the liver or spleen [158]. The questions are repeatedly quantified and discussed in contemporary reviews and meta-analyses of nanomedicine [159,160,161].

### 4.1. Biochemical and Pharmacological Radiosensitizers

#### 4.1.1. Cell-Cycle Checkpoint/Kinase Control

Radiosensitivity is strongly influenced by cell-cycle phase: cells are most sensitive in G2/M and more resistant in late S phase [143,162]. Cyclin-dependent kinases (CDKs) regulate these transitions while also coordinating the DNA repair capacity. Inhibition of CDK4/6 (e.g., palbociclib, ribociclib, abemaciclib) leads to G1 arrest and reduces homologous recombination efficiency, thereby impairing repair of IR-induced DSBs. In breast cancer models, CDK4/6 blockade increased persistence of γH2AX foci, augmented apoptosis, and enhanced clonogenic radiosensitivity [163,164]. Early clinical data suggest that combining CDK4/6 inhibitors with radiotherapy is feasible, although toxicity profiles (particularly hematologic) require careful monitoring [165,166,167,168].

#### 4.1.2. Redox Modulation

Ionizing radiation induces ROS that damage DNA, proteins, and lipids. Tumor cells often counterbalance this by upregulating antioxidant defenses (e.g., glutathione, superoxide dismutase) [169,170,171]. Radiosensitizers in this class act by overwhelming antioxidant defenses. Disulfiram, an aldehyde dehydrogenase inhibitor, forms copper complexes that catalyze ROS production, inhibit the proteasome, and induce apoptosis; preclinical studies confirm enhanced radiosensitivity when combined with IR [172,173,174]. Other strategies include pharmacologic glutathione depletion (e.g., buthionine sulfoximine) or inhibition of NADPH supply, both of which sensitize tumors to RT [175,176,177].

#### 4.1.3. Metabolic Disruption

Radiation response is coupled to tumor metabolism: efficient glycolysis and mitochondrial function supply ATP and NADPH needed for DNA repair and antioxidant defence [178,179]. Pharmacologic inhibition of glycolysis (e.g., 2-deoxy-D-glucose, LDH inhibitors) or blockade of mitochondrial oxidative phosphorylation (e.g., metformin, complex I inhibitors) deprives tumor cells of energy and reduces their redox potential. 2-Deoxy-D-glucose has been evaluated as a glycolytic radiosensitizer in early clinical studies with RT [180]. Metformin (complex I inhibition) has preclinical support for radiosensitizing breast cancer (e.g., MCF-7) via ROS/thioredoxin effects and is being explored as an adjuvant in RT [181]. This metabolic stress exacerbates IR-induced DNA lesions by limiting the activity of repair proteins and increasing ROS accumulation [182].

#### 4.1.4. Immunomodulation

Radiation can act as an in situ vaccine by inducing immunogenic cell death, releasing tumor antigens, and activating cytosolic DNA sensing through the cGAS–STING pathway. However, tumor-intrinsic immunosuppressive signals often dull this effect. Immune checkpoint inhibitors, or the checkpoint blockade (anti-PD-1/PD-L1, anti-CTLA-4), restore T-cell activity, and when combined with RT, synergistically amplify antitumor immunity [103,183]. The pharmacological radiosensitization here is indirect: RT primes the immune response, while checkpoint blockade sustains and expands it, converting local RT into a systemic abscopal effect [184,185]. Early-phase trials in lung cancers have shown encouraging response rates, although the optimal sequencing and fractionation strategies remain under study [186,187,188].

### 4.2. Direct Targeting of the DNA Damage Response

Due to the predominantly induced DSBs by the IR, inhibiting repair pathways is a direct radiosensitization strategy. The strategies range from selective inhibition of the NHEJ pathway to dual or multiple pathway inhibition. The selective DNA-PK inhibitor AZD7648 sensitizes tumors to RT in vitro and in vivo (including immunogenic cell death and CD8^+^/type-I IFN-dependent effects) [122,126]. In breast cancer specifically, NU7441 delayed DSB repair and increased radiosensitivity in MCF-7 and MDA-MB-231 cells [111]. Early clinical testing shows peposertib (M3814) can be combined with RT +/− cisplatin in thoracic/HNSCC settings (phase I) [117]. The dual ATM/DNA-PK inhibitor XRD-0394 enhances radiosensitization and potentiates PARP/topo-I inhibitors in preclinical studies [189]. CC-115, a dual DNA-PK/mTOR inhibitor, illustrates concurrent impairment of DSB repair and survival/translation signaling and has completed first-in-human testing [119]. DNA-PK inhibitors have shown promise as radiosensitizers, potentially enabling effective treatment with lower radiation doses. Preclinical studies indicate that adding a DNA-PK inhibitor can greatly reduce the radiation needed for tumor control. For example, NU7441 increased cancer cell sensitivity to ionizing radiation by roughly 4–12-fold in vitro [111], implying a much lower radiation dose could achieve the same cytotoxic potential. Early clinical data echo these findings: the oral DNA-PK inhibitor peposertib (M3814) was a potent radiosensitizer in a phase 1 trial, investigators suggesting that using peposertib might allow reduced radiation fractions or total dose while still maintaining similar efficacy [117]. Likewise, newer agents such as AZD7648 and VX-984 have demonstrated enhanced tumor responses to radiation in preclinical models, supporting the possibility of lower-dose radiotherapy regimens when combined with DNA-PK blockade [108]. These findings cover various tumor types and contexts, while further studies are still needed, they underscore that DNA-PK inhibition can significantly amplify the effects of radiation—a strategy that could allow for lower radiation doses to be used without compromising anti-tumor activity.

Although HR is maximally active during the S and G2 phases of the cell cycle, it is a kinetically slow process. In contrast, Non-Homologous End Joining (NHEJ) is a rapid-response mechanism that remains the dominant pathway for repairing ionizing radiation (IR)-induced double-strand breaks (DSBs) throughout all cell cycle phases [3]. It was indicated that NHEJ repairs approximately 75–80% of IR-induced DSBs even in G2 phase [190], largely because it engages minutes after irradiation, whereas HR requires extensive end-resection and operates over hours. Radioresistant tumors tend to preserve or upregulate this fast repair mechanism; therefore, inhibition of the NHEJ pathway (e.g., via DNA-PK inhibitors) remains a potent radiosensitizing strategy in rapidly dividing cancer cells by blocking this primary, fast-repair avenue, functionally isolating and overwhelming the slower HR reserve, driving accumulation of unrepaired DSBs and forcing cells into mitotic catastrophe.

DNA-PK is indispensable for classical NHEJ—the dominant DSBs repair route post-irradiation—while mTOR provides metabolic and translational support that sustains repair, proliferation, and survival under genotoxic stress. Reviews detail functional crosstalk between mTOR and DDR, providing a mechanistic basis for combined targeting to enhance radiosensitization and counter radioresistance [57,191]. Wilson et al. report a correlation observed in cervical carcinoma biopsies, where tumors with low Ku70 protein expression showed higher radiosensitivity and were associated with significantly better patient survival [192].

Key challenges for translation include biomarker-based patient selection (e.g., NHEJ proficiency, DNA-PK expression, hypoxia signatures), sparing normal tissue while maximizing tumor radiosensitization, and rational integration with immunotherapy and targeted agents. Implementation of radiosensitization techniques in combination with therapeutic and theranostic radioisotopes can increase the efficiency of the treatment by increasing the debilitating effects of decay emissions in cancerous cells in targeted radionuclide therapy [174,175,176]. Continued emphasis on prospective biomarker strategies and careful combination scheduling is essential to convert robust preclinical radiosensitization into durable clinical benefit [139].

Despite the efficacy of DNA-PK inhibitors in preclinical models, tumor cells can develop complicated resistance mechanisms. Intratumoral heterogeneity in DNA repair reliance means that only subpopulations of tumor cells may be highly NHEJ-dependent, allowing other clones (with greater HR or alt-NHEJ capacity) to survive NHEJ blockade [56]. Moreover, inhibiting DNA-PK can provoke compensatory pathway activation: suppression of DNA-PK–mediated repair causes a surge in alternative DSB repair processes, with studies showing that DNA-PKcs inhibition reduces NHEJ activity by ~94% while concurrently upregulating homologous recombination and polymerase θ–mediated end-joining (alt-NHEJ) over 3-fold [193]. Cells may also restore upstream signalling; loss of DNA-PK function leads to hyperactivation of ATM kinase and an amplified p53 DNA-damage response [194,195]. Critically, p53-deficient tumors bypass this fail-safe—DNA-PK inhibition fails to induce apoptosis in p53-null cells, which cannot execute the ATM/p53-dependent damage response [195]. Other escape mechanisms include drug efflux: for instance, overexpression of the ABCG2 transporter reduces intracellular levels of the DNA-PK inhibitor CC-115, conferring resistance, considering that CC-115 is a substrate of both ABCG2 and ABCB1 [196]. Together, these adaptations enable cancer cells to withstand DNA-PK–targeted therapies, underscoring the importance of overcoming therapeutic resistance by utilizing mutually potentiating therapeutic combinations.

### 4.3. Safety, Selectivity and Normal Tissue Tolerance

A major concern regarding DNA-PK inhibition is the potential for indiscriminate radiosensitization, leading to severe healthy tissue toxicity (e.g., fibrosis or necrosis). However, emerging clinical data suggest a manageable safety profile, driven by biological selectivity. Tumoral selectivity is largely achieved through “synthetic lethality” [197]: normal tissues generally possess intact cell cycle checkpoints, as p53-mediated G1 arrest, and functional alternative repair pathways (such as HR in S/G2 phase). In contrast, many radioresistant tumors harbor defects in p53, ATM, or redundant repair mechanisms, rendering them exclusively dependent on the rapid NHEJ pathway to survive radiation-induced breaks [138].

Furthermore, off-target toxicity—historically a hurdle due to the structural homology between DNA-PK and PI3K—has been mitigated by “next-generation” inhibitors with high kinase selectivity (e.g., AZD7648, M3814) [126]. Clinical trials combining these agents with radiotherapy have reported dose-limiting toxicities that are primarily hematological or gastrointestinal, rather than severe synergistic radiation toxicity in normal tissues. For example, the combination of peposertib with palliative radiotherapy demonstrated that while DNA-PK was inhibited in surrogate tissues (peripheral blood mononuclear cells), the regimen did not result in unexpected grade 3/4 radiation-associated toxicities, suggesting that normal tissues can tolerate transient NHEJ suppression during fractionated treatment [117].

## 5. Conclusions

The intricate orchestration of DNA repair mechanisms—anchored by NHEJ, HR, DNA-PK, and mTOR signaling—ensures the preservation of genomic stability against persistent endogenous and exogenous threats. Their dynamic crosstalk illustrates not only the molecular elegance of the DNA damage response but also the vulnerabilities that can be therapeutically exploited. Dysregulation of these pathways underlies oncogenesis, therapy resistance, and altered metabolic states, while the targeted modulation of these pathways has already shown promise in sensitizing tumors to radiation and chemotherapy, underscoring the pressing need for innovative approaches that modulate DNA repair.

Emerging evidence suggests that selectively targeting these mechanisms can profoundly influence cellular sensitivity to genotoxic stress, opening possibilities for refined therapeutic strategies that extend beyond conventional cytotoxic paradigms. Yet, despite significant advances, the field still grapples with critical questions: how to exploit the best differential DNA repair capacities among tumor subtypes, how to maximize radiosensitization while sparing normal tissues, and how to translate mechanistic insights into durable clinical benefits. Filling these gaps is essential for improving therapeutic accuracy in oncology and sets the groundwork for the next wave of experimental and translational research.

The future landscape of DNA-PK inhibition will likely move from broad chemosensitization to precision medicine strategies that exploit specific tumor vulnerabilities and immune potentiation [198]. Moving further, future trials must integrate predictive biomarkers to identify the patients most likely to benefit from it. Preclinical data suggest that tumors with defects in ATM or p53 are synthetically lethal with DNA-PK inhibition because they lack functional G1/S checkpoints, rendering them exclusively dependent on NHEJ for survival [116]. Furthermore, emerging evidence indicates that DNA-PK inhibition can potentiate antitumor immune activity. By preventing DSB repair, these agents trigger the activation of the Cyclic GMP-AMP Synthase–Stimulator of Interferon Genes (cGAS-STING) by raising the concentration of cytosolic damaged DNA (dsDNA). Once activated, the STING-cGAMP complex recruits and activates TANK-binding kinase 1 (TBK1) which further phosphorylates Interferon Regulatory Factor 3 (IRF3). IRF3, in turn, translocates into the nucleus and promotes pro-inflammatory chemokines transcription [199]. Looking ahead, as radioligand therapies will expand, resistance mechanisms involving DNA repair will become position as a growing hurdle. DNA-PK inhibitors might offer a strategic partner for Targeted Radiotherapy (TRT), potentially enhancing its efficacy [51].

Implementation of DNA-PK inhibitors in the context of stratifying therapeutic decisions by a tumor’s NHEJ dependency offers a rational path to enhance radiotherapy efficacy while minimizing unnecessary toxicity. Functional profiling of DSB-repair competence, paired with DNA-PK–targeted radiosensitization, before other genotoxic treatments (therapeutic and theranostic radiopharmaceuticals [200,201,202] and DNA-damaging chemotherapeutics) can increase long-term survival and the patients’ recovery. Ultimately, integrating biomarker-based selection into prospective trials offers a practical path to translate mechanistic insights into personalized radiotherapy.

## Figures and Tables

**Table 1:** DNA-PK inhibitors.

Name	Molecule	Molecular Weight	Target	IC50
NU7441 (KU-57788)	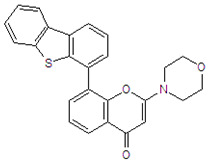	413.49	DNA-PK/PI3K/mTOR	14 nM/5 μM/1.7 μM
M3814-Peposertib	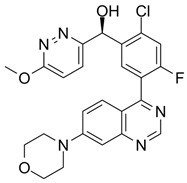	481.91	DNA-PK	3 nM
CC-115	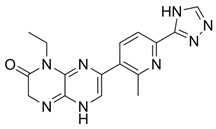	336.35	DNA-PK/mTOR	0.013 μM/0.021 μM
AZD7648	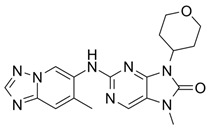	380.4	DNA-PK	0.6 nM
VX-984 (M9831)	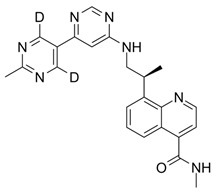	415.49	DNA-PK	88 ± 64 nM
PIK-75 HCl	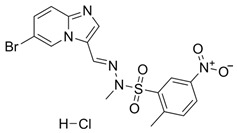	488.74	P110a/DNA-PK	5.8 nM/2 nM

## Data Availability

No new data were created or analyzed in this study.

## Abbreviations

The following abbreviations are used in this manuscript:

4E-BP1	Eukaryotic translation initiation factor 4E-binding protein 1
53BP1	p53-binding protein 1
ABCB2	Transporter Associated with Antigen Processing 1
ABCG1	ATP-binding cassette sub-family G member 1
ADP	Adenosine diphosphate
AKT	Protein kinase B
alt-NHEJ	Polymerase θ–mediated end-joining
AML	Acute myeloid leukemia
AMP	Adenosine monophosphate
ATM	Ataxia-Telangiectasia Mutated
ATP	Adenosine triphosphate
BRCA1	Breast Cancer gene 1
BRCA2	Breast Cancer gene 2
Cas9	CRISPR-associated protein 9
CD8^+^	Cytotoxic T lymphocytes
Cdk1	Cyclin-dependent kinase 1
CDK4	Cyclin-dependent kinase 4
CDK6	Cyclin-dependent kinase 6
CDKs	Cyclin-dependent kinases
cGAS-STING	Cyclic GMP-AMP Synthase–Stimulator of Interferon Genes
cNHEJ	Canonical NHEJ
CRISPR-Cas9	Clustered Regularly Interspaced Short Palindromic Repeats
cryo-EM	Cryo-electron-microscopy
ctDNA	Circulating Tumor DNA
DSB	DNA double-strand break
DDR	DNA damage response
DDRNAs	DNA damage response RNAs
dilncRNAs	Damage-induced long non-coding RNAs
DNA	Deoxyribonucleic acid
DNA-PK	DNA-dependent protein kinase
EGF	Epidermal growth factor
FKBP12	FK506-binding protein 12
GMP	Guanosine monophosphate
HDR	Homology-directed-repair
HIF-1α	Hypoxia-Inducible Factor 1-alpha
HIFs	Hypoxia-inducible factors
HNSCC	Squamous cell carcinoma of the head and neck
HR	Homologous recombination
IC_50_	Half-maximal inhibitory concentration
IFN	Interferon
IGF	Insulin-like growth factor I
IL-6	Interleukin-6
IR	Ionizing radiation
IRF3	Interferon Regulatory Factor 3
JNK	c-Jun N-terminal kinase
Ku70	ATP-dependent DNA helicase 2 subunit 1
Ku80	X-ray repair cross-complementing protein 5
LDH	Lactate dehydrogenase
Lig4	Ligase IV
MCF-7	Human breast cancer cell line
MCL-1	Myeloid cell leukemia-1
MDA-MB-231	Human breast cancer cell line
MRN	MRE11-RAD50-NBS1
MRE11	Meiotic recombination 11
mRNA	Messenger RNA
MSH6	MutS Homolog 6
mTOR	Mammalian Target of Rapamycin
mTORC1	mTOR Complex 1
mTORC2	mTOR Complex 2
NADPH	Nicotinamide adenine dinucleotide phosphate, reduced form
NBS1	Nibrin
NBTXR3	Hafnium oxide nanoparticles cancer therapy
NF-κB	Nuclear factor kappa-light-chain-enhancer of activated B cells
NHEJ	Non-homologous end joining
NSC11	Glioblastoma cancer stem cell line
NSCLC	Non-small cell lung cancer
p110α	Isoform of the PI3K catalityc subunit
p53	Tumor protein p53
PARP	Poly(ADP-ribose) polymerase
PAXX	Paralog of XRCC4 and XLF
PDK1	3-phosphoinositide-dependent kinase 1
PH/PHIP	Pleckstrin homology
PI3K	Phosphoinositide 3-kinase
PIKK	Phosphatidylinositol-3-kinase-related protein kinase
PIP2	Phosphatidylinositol 4,5-bisphosphate
PIP3	Phosphatidylinositol 3,4,5-trisphosphate
PKB	Protein Kinase B
Pol X	DNA Polymerase X family
Pol α	DNA polymerase alpha
Pol λ	DNA polymerase lambda
Pol μ	DNA polymerase mu
PRKDC	Protein kinase, DNA-activated, catalytic subunit gene
PSA	Prostate-specific antigen
PTEN	Phosphatase and Tensin Homolog proteins
RAD51	Radiation Defective 51
RNA	Ribonucleic acid
RNAPII	RNA polymerase II
ROS	Reactive oxygen species
RPS6	Ribosomal Protein S6
RT	Radiotherapy
RTK	Receptor tyrosine kinase
S6K	S6 kinase beta
STING-cGAMP	Stimulator of interferon genes
TANK	TRAF-associated NF-κB activator
TBK1	TANK-binding kinase 1
TH-302	Evofosfamide
TNF-α	Tumor necrosis factor
topo-I	Topoisomerase I
TRAF	Tumor Necrosis Factor Receptor-Associated Factor
TRT	Targeted Radiotherapy
U251	Human glioblastoma multiforme
VEGF	Vascular endothelial growth factor
XLF	XRCC4-like factor
XRCC4	X-ray repair cross-complementing protein 4
XRCC7	X-ray repair cross-complementing protein 7
γH2AX	Gamma-H2A histone family member X
